# Comparison of Dietary Control and Atorvastatin on High Fat Diet Induced Hepatic Steatosis and Hyperlipidemia in Rats

**DOI:** 10.1186/1476-511X-10-23

**Published:** 2011-01-26

**Authors:** Guiyuan Ji, Xihong Zhao, Liang Leng, Peiyi Liu, Zhuoqin Jiang

**Affiliations:** 1 Department of Nutrition, School of Public Health, Sun Yat-Sen University, Guangzhou, 510080, China; 2 Key Laboratory for Green Chemical Process of Ministry of Education, School of Chemical Engineering and Pharmacy, Wuhan Institute of Technology, Wuhan 430073, China

## Abstract

**Background:**

Treatment with atorvastatin (ATO) or dietary control has been demonstrated to benefit patients with non-alcoholic fatty liver disease (NAFLD) and hyperlipidemia. However, little is known on whether combination of dietary control and ATO treatment could enhance the therapeutic effect.

**Methods:**

We employed a rat model of NAFLD to examine the therapeutic efficacy of dietary control and/or ATO treatment. Sprague-Dawley rats were fed with normal chow diet as normal controls or with high fat diet (HFD) for 12 weeks to establish NAFLD. The NAFLD rats were randomized and continually fed with HFD, with normal chow diet, with HFD and treated with 30 mg/kg of ATO or with normal chow diet and treated with the same dose of ATO for 8 weeks. Subsequently, the rats were sacrificed and the serum lipids, aminotranferase, hepatic lipids, and liver pathology were characterized. The relative levels of fatty acid synthesis and β-oxidation gene expression in hepatic tissues were measured by quantitative real-time polymerase chain reaction (qRT-PCR). Hepatic expression of hydroxy-3-methylglutaryl coenzyme A (HMG-CoA) reductase was determined by Western blot assay.

**Results:**

While continual feeding with HFD deteriorated NAFLD and hyperlipidemia, treatment with dietary control, ATO or ATO with dietary control effectively improved serum and liver lipid metabolism and liver function. In comparison with ATO treatment, dietary control or combined with ATO treatment significantly reduced the liver weight and attenuated the HFD-induced hyperlipidemia and liver steatosis in rats. Compared to ATO treatment or dietary control, combination of ATO and dietary control significantly reduced the levels of serum total cholesterol and low density lipoprotein cholesterol (LDL-C). However, the combination therapy did not significantly improve triglyceride and free fatty acid metabolism, hepatic steatosis, and liver function, as compared with dietary control alone.

**Conclusions:**

ATO treatment effectively improved NAFLD-related hyperlipidemia and inhibited liver steatosis, accompanied by modulating the expression of genes for regulating lipid metabolism. ATO enhanced the effect of dietary control on reducing the levels of serum total cholesterol and LDL-C, but not triglyceride, free fatty acid and hepatic steatosis in HFD-induced fatty liver and hyperlipidemia in rats.

## Background

Non-alcoholic fatty liver disease (NAFLD) is a common hepatic disease, and pathologically, it can display as simple steatosis, non-alcoholic steatohepatitis (NASH), and eventually progress to cirrhosis, an end-stage liver disease[1]. The prevalence of NAFLD ranges from 6% to 14% in different populations [2]. The median prevalence of ultrasonographic steatosis in Chinese populations is about 10%, but varies from 1% to more than 30%[3]. Notably, 1%-5% of patients with simple steatosis can eventually develop actual cirrhosis, and 10% to 15% of patients with NASH can progress to cirrhosis and even to hepatocellular carcinoma[4,5].

The pathogenic process of NAFLD is not well understood and effective therapy for NAFLD has not been established. Currently, treatment for NAFLD is generally dependent on gradual loss of body weight and change in lifestyle. However, these strategies have poor compliance in many patients, and whether these strategies are still beneficial for patients with advanced disease is unknown. Numerous efforts have been directed at exploring new therapeutic reagents. Insulin receptor sensitizing agents and antioxidants have been tested for the treatment of NAFLD[6]. However, their therapeutic efficacy and clinical safety remain to be established. Recently, several studies reveal that treatment with atorvastatin (ATO) is effective and safe for patients with NAFLD or NASH with hyperlipidemia[7,8]. However, whether the efficacy of ATO treatment is better than dietary control and whether ATO can synergize with dietary control to enhance the therapeutic efficacy for NAFLD have not been explored.

This study aimed at examining the therapeutic efficacy of dietary control combined with ATO treatment in a rat model of NAFLD with hyperlipidemia and at exploring potential mechanism(s) underlying the therapeutic effect of dietary control and/or ATO treatment on inhibiting the high fat diet (HFD)-induced hyperlipidemia and liver steatosis.

## Results

### Establishment of rat model of NAFLD

**Figure 1:**
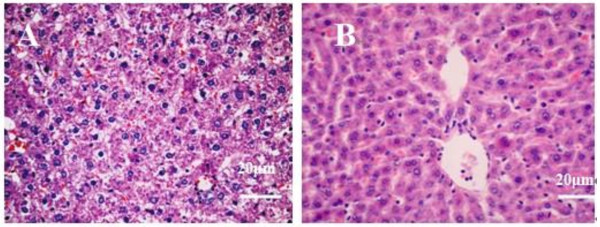
**Histological analysis of the rat livers**. Male SD rats were fed with normal chow diet (normal control) or with HFD for 12 weeks, and their liver tissue sections were stained with H&E staining, followed by examining under a light microscope. Data shown are representative images (magnification 40x) of normal control and NAFLD rats (n = 6 per group) from two separate experiments. (A) HFD-fed NAFLD rats; (B) Normal control rats

Male SD rats were fed with HFD or normal chow diet for 12 weeks, and six rats were randomly chosen from each group for liver histopathology. Hepatocytes in the HFD-fed rat liver tissues displayed different sizes of lipid droplets in the cytoplasm (Figure 1A). However, the fatty liver disease-related characteristic was absent in the liver tissues of normal control rats (Figure 1B). Hence, feeding with HFD for 12 weeks induced NAFLD in rats.

### Dietary control and ATO treatment reduced the liver weight in NAFLD rats

Following treatment with ATO for 8 weeks, the body and liver weights as well as the ratios of liver to body weights in each group of rats were measured (Table 1). There was no significant difference in the body weight among these groups of rats (*p *> 0.05). In contrast, the liver weight and the ratios of liver to body weights in the model group were significantly higher than that of other groups (*p *< 0.05 for all). The liver weights and the ratios of liver to body weights between the DC and DCA groups were not significantly different (*p *> 0.05), but they were significantly lower than that of the ATO group (*p *< 0.05, *p *< 0.05). Apparently, continual feeding with HFD deteriorated the hepatic steatosis and predominately increased the liver weights, while dietary control by feeding with normal chow diet or combined with ATO treatment inhibited the progression of hepatic steatosis and the gain in liver weights in NAFLD rats.

**Table 1:** The body weights, liver weights and the ratios of liver to body weights in rats

Group	Liver weight (g)	Body weight (g)	Liver/Body (%)
Normal	12.48 ± 0.91	547.72 ± 25.95	2.28 ± 0.12
Model	27.00 ± 3.56^†^	542.14 ± 34.40	4.98 ± 0.58^†^
DC	13.80 ± 1.88*^‡^	509.11 ± 48.39	2.71 ± 0.30*^‡^
DCA	12.81 ± 1.16*^‡^	520.23 ± 28.97	2.46 ± 0.19*^‡^
ATO	20.92 ± 2.45*	512.11 ± 51.87	4.11 ± 0.56*

Normal: The rats received normal chow diets throughout the experimental period; Model: The rats fed with high fat diet (HFD) continually for 20 weeks. DC: Dietary control (rats were fed with HFD for 12 weeks, and then fed with normal diet for 8 weeks); ATO (atorvastatin): Treatment with ATO alone (rats were fed with HFD for 12 weeks, and then fed with HFD and atorvastatin for 8 weeks); DCA: Dietary control combined with ATO treatment (rats were fed with HFD for 12 weeks, and then normal diet with atorvastatin for 8 weeks). Values are mean ± SD, n = 8 per group. ^†^*p *< 0.05 versus the normal group; *p < 0.05 versus the model group; ^‡ ^*p *< 0.05 versus the ATO group.

### Dietary control and ATO treatment attenuated the HFD-induced hepatic steatosis

**Figure 2:**
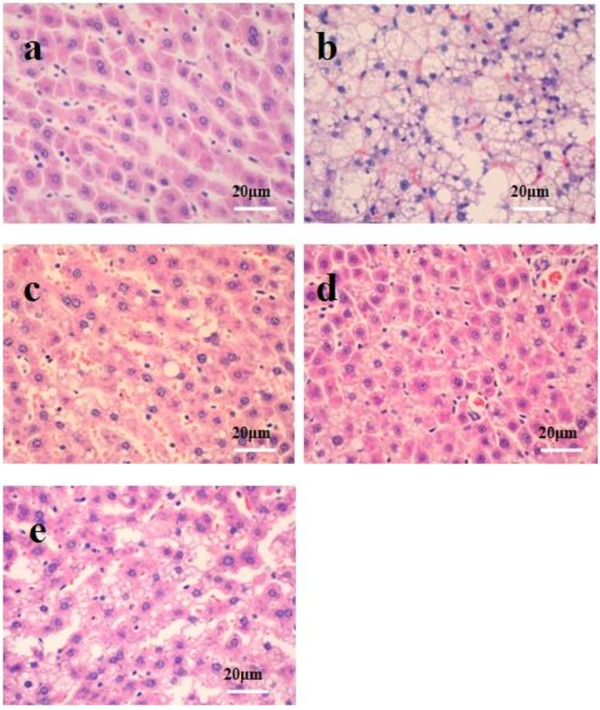
**Pathogenic degrees of the rat livers**. Histological analysis of the rat livers. NAFLD rats were randomized and fed with HFD (Model), with HFD and treated p.o with 30 mg/Kg body weight of ATO daily (ATO), with normal chow diet (DC), with normal chow diet and treated with the same dose of ATO (DCA) for 8 weeks. Healthy rats were fed continually with normal chow diet and used as normal controls (Normal). Their liver tissue sections were stained with H&E and examined under a light microscope. Data shown are representative images (magnification 40x) of each group (n = 8 per group) from four independent experiments and control and experimental rats were analyzed simultaneously. (a) Normal control group; (b) The model group; (c) The DC group; (d) The DCA group; and (e) The ATO group.

**Figure 3:**
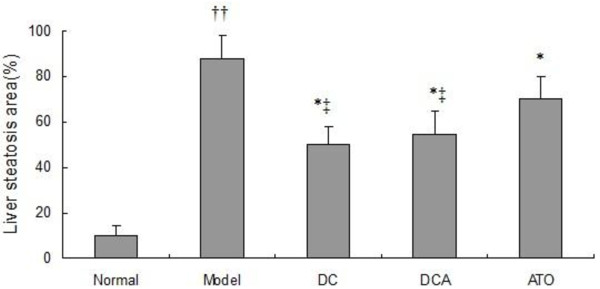
**Scores of hepatic steatosis of rat livers**. The scores were determined, according to the the percentage of hepatocytes containing lipid droplets. Data are expressed as mean ± SD of each group (n = 8 per group) and determined by a pathologist in a blinded fashion. ^††^*p *< 0.01 versus the normal group; *p < 0.05 versus the model group; ^‡ ^*p *< 0.05 versus the ATO group.

After being stained with H&E, the degrees of hepatic steatosis were examined. While there was no obvious steatosis in the liver of the normal group of rats, different degrees of liver steatosis were observed in other group rats (Figure 2). The area of hepatic steatosis in the DC and DCA groups decreased remarkably when compared with that in the model group, however, there was no significant difference in the score of liver steatosis between the DC and DCA groups. Further analysis revealed that the score of hepatic steatosis in the ATO group was significantly reduced, as compared with that in the model group, however, they remained significantly higher than that of the DC or DCA group (Figure 3).

Similarly, the levels of hepatic TC and TG in the model group were significantly higher than that of the normal control, DC and DCA groups, but there was no obvious difference between the DC and DCA groups. The levels of hepatic TC in the ATO group remained significantly higher than that of the DC and DCA groups (Table 2).

**Table 2:** The levels of serum animotransferases and hepatic lipids in rats

Group	Serum ALT (U/L)	Serum AST (U/L)	Hepatic TC (mg/g)	Hepatic TG (mg/g)
Normal	30.30 ± 4.79	69.60 ± 7.78	0.94 ± 0.21	1.61 ± 0.82
Model	79.30 ± 22.23^††^	135.20 ± 26.52^††^	6.33 ± 1.59^††^	3.51 ± 0.77^††^
DC	42.70 ± 12.72**	79.10 ± 13.4**^‡^	4.19 ± 1.15**^‡^	2.29 ± 0.95**
DCA	40.60 ± 5.50**	73.6 ± 11.26**^‡^	4.26 ± 1.02**^‡^	2.10 ± 0.79**
ATO	43.40 ± 7.41**	100.70 ± 6.04*	4.90 ± 0.27	2.53 ± 0.65*

Values are mean ± SD, n = 8 per group. DC: dietary control; ATO: atorvastatin; DCA, dietary control combined with ATO. ^††^*p *< 0.01 versus the the normal group; **p < 0.01 versus the model group; *p < 0.05 versus the model group; ^‡^*p *< 0.05 versus the ATO group.

To determine whether dietary control and ATO treatment could mitigate the HFD-induced liver injury, the concentrations of serum ALT and AST in different groups of rats were examined. The concentrations of serum ALT and AST in the model group were significantly higher than that of the normal control, DC, DCA and ATO groups. While the levels of serum ALT and AST were comparable between the DC and DCA groups. The level of serum AST, but not ALT, in the ATO group were significantly higher than that of the DC and DCA groups (Table 2).

### Dietary control and ATO treatment improved serum lipid profiles

As shown in Table 3, there was no significant difference in the level of serum HDL-C among these groups of rats. The concentrations of serum TC, TG, and LDL-C in the model group were significantly higher than those of other groups (*p *= 0.002, *p *= 0.001, and *p *= 0.002 respectively). There was no significant difference in the levels of serum TC, TG, and LDL-C between the DC and ATO group rats. Interestingly, the levels of serum TC and LDL-C, but not TG in the DCA group was significantly lower than those of the DC and ATO. Finally, analysis of serum FFA revealed that the levels of serum FFA in the model group were significantly higher than that of other groups, while no significant difference was detected in the normal control, DC, DCA, and ATO groups of rats. These data indicated that combination of dietary control and ATO treatment improved lipid profiles in the HFD-fed rats.

**Table 3:** The levels of serum lipids in rats

Group	TC (mmol/L)	TG (mmol/L)	LDL-C(mmol/L)	HDL-C(mmol/L)	FFA(μmmol/L)
Normal	1.45 ± 0.14	0.68 ± 0.14	0.24 ± 0.07	0.82 ± 0.14	50.14 ± 11.10
Model	4.36 ± 1.70^††^	0.91 ± 0.10^††^	3.82 ± 1.88^††^	0.83 ± 0.18	63.55 ± 13.68^†^
DC	2.15 ± 0.23**	0.70 ± 0.22**	1.28 ± 0.08**	0.82 ± 0.16	42.66 ± 11.89*
DCA	1.47 ± 0.19**^‡^	0.72 ± 0.13**	0.33 ± 0.10**^‡^	0.83 ± 0.16	45.33 ± 9.76*
ATO	2.35 ± 0.40*	0.75 ± 0.26**	1.30 ± 0.44**	0.82 ± 0.13	50.40 ± 13.38*

DC: dietary control; ATO: atorvastatin; DCA, dietary control combined with ATO. Values are mean ± SD, n = 8. ^††^*p *< 0.01 versus the normal group; **p < 0.01 versus the model group; *p < 0.05 versus to model group; ^‡^*p *< 0.05 versus the ATO group.

### Effect of dietary control and ATO treatment on the transcription of the PPARα and SREBP-1c-related genes in the liver

**Figure 4:**
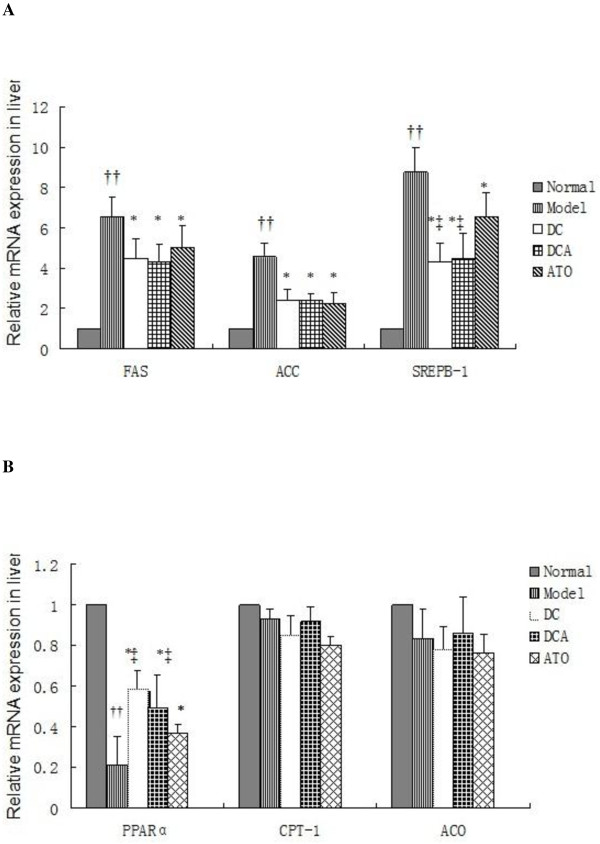
**The expression of lipid metabolic regulators in the liver**. (A) The expression of hepatic SREBP-1c and related genes. The relative levels of hepatic SREBP-1c, FAS, and ACC mRNA transcripts to control GAPDH were determined by quantitative RT-PCR. Data are expressed as mean ± SD of each group (n = 8 per group) from three independent experiments and control, and experimental rat livers were simultaneously analyzed. The value of the normal control group was designated as 1 for each gene. (B) The expression of PPARα and related genes. The relative levels of PPARα, CPT-1, and ACO mRNA transcripts to control GAPDH were determined by quantitative RT-PCR. The value of the normal control group was designated as 1 for each gene. ^††^*p *< 0.01 versus the normal group; *p < 0.05 versus the model group; ^‡^*p *< 0.05 versus the ATO group.

To explore the mechanisms underlying the effect of dietary control and ATO treatment, the relative levels of PPARα and SREBP-1c-related gene transcripts in the liver were determined by qRT-PCR. The relative levels of SREPB-1c and its target FAS and ACC mRNA transcripts in the model group were 4-8 fold higher than that of the normal control group (Figure 4A). In contrast, the relative levels of those transcripts in the DC, DCA, and ATO groups were reduced by 25-50%, as compared with that in the model group, although they were still significantly higher than that of the normal control group. While there was no significant difference in the relative levels of FAS and ACC among the DC, DCA, and ATO groups, the relative levels of SREBP-1c mRNA transcripts in the ATO group were higher than that of the DC and DCA groups (*p *< 0.05). Apparently, dietary control and ATO treatment inhibited the HFD-induced transcription of these genes in rats. Further analysis revealed that the relative levels of PPARα mRNA transcripts, but not CPT-1 and ACO, in HFD-fed rats were significantly lower than that in the normal controls(*p *< 0.01) (Figure 4B). Furthermore, the relative levels of PPARα mRNA transcripts in the model group were significantly lower than that of the DC, DCA, and ATO groups of rats (*p *< 0.05, *p *< 0.05, *p *< 0.05), while the relative levels of PPARα mRNA transcripts in the ATO group were also significantly lower than that of the DC and DCA groups of rats (*p *< 0.05, *p *< 0.05). These data indicated that feeding with HFD significantly inhibited the PPARα transcription, but did not affect the expression of other lipid-oxidative genes tested. Dietary control and ATO treatment appeared to antagonize the effect of HFD in rats.

### Effect of dietary control and ATO treatment on the expression of HMG-CoA reductase in the liver

**Figure 5:**
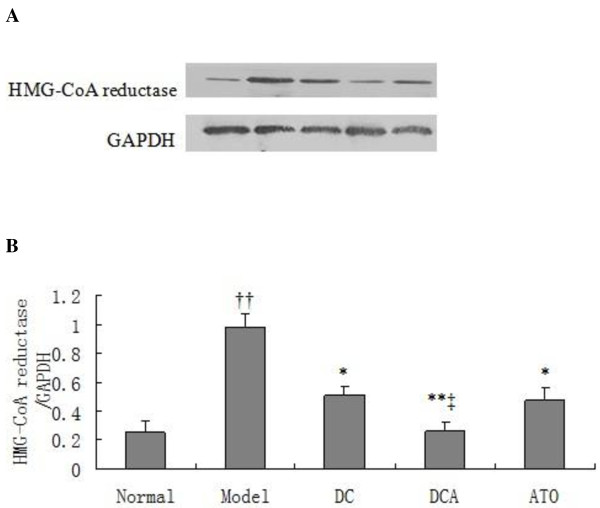
**Western blot analysis of hepatic HMG-CoA reductase in rats**. The relative levels of hepatic HMG-CoA reductase to control GAPDH in different groups of rats were determined by Western blot assays using specific antibodies. (A) Western blot analysis. Data shown are representative images. (B) Quantitative analysis. Data are expressed as means ± SD of each group, (n = 8) from three separate experiments. ^††^*p *< 0.01 versus the normal group; *p < 0.05 versus the model group; **p < 0.01 versus the model group; ^‡^*p *< 0.05 versus the ATO group.

HMG-CoA reductase is most abundantly expressed in the liver, and plays a central role in the regulation of plasma cholesterol concentration[9]. ATO is an inhibitior of the HMG-CoA reductase and can inhibit cholesterol synthesis. As shown in Figure 5, the relative expression of HMG-CoA reductase in the liver of the model of group rats was 3.9-fold higher than that of the normal control (*p *= 0.001). However, its expression was significantly reduced by 48%(*p *< 0.05), 73% (*p *< 0.01) and 52% (*p *< 0.05) in the DC, DCA and ATO group of rats, respectively, More importantly, the relative levels of HMG-CoA reductase in the livers of DCA group of rats were significantly lower than that of the ATO groups of rats (*p *< 0.05). Moreover, the levels of HMG-CoA reductase in DCA group of rats decreased more significantly than those in DC.

## Discussion

NAFLD is a common chronic liver disease worldwide and its incidence is increasing in developed countries [10]. Over-consumption of high calories of foods, particularly HFD, is crucial for the development of NASH and NAFLD. Conceivably, control of diet is important for the prevention and intervention of NASH. Dietary control and lifestyle modification appear to be an effective therapeutic strategy for intervention of NAFLD in individuals with obese and insulin resistance [11,12]. Recent studies have shown that treatment with ATO also benefits patients with NASH and NAFLD[7,8]. In this study, we compared the efficacy of dietary control and/or ATO treatment on the HFD-induced hepatic steatosis and hyperlipidemia in rats. We found that dietary control or combined with ATO treatment for 8 weeks significantly inhibited the HFD-induced liver weights and reduced the ratios of liver to body weights in the NAFLD rats. However, we did not observe significant difference in the body weights among those groups of rats, consistent with previous reports in this model of rats[13,14]. The reason may be that a high fat diet may induce anorexia in rats [15,16]. Furthermore, dietary control or combined with ATO treatment mitigated the HFD-induced hepatic steatosis, which was associated with the improvement of liver and systemic lipid profiles and function, leading to reduction in the severity of hyperlipidemia in NAFLD rats. Therefore, our data support the notion that dietary control and personal lifestyle modifications are critical for the control of hyperlipidemia and associated NAFLD. However, whether dietary control for a longer period could prevent the development of NAFLD-related liver fibrosis and dietary control could improve lipid metabolism in advanced NAFLD, remain to be further investigated.

Surprisingly, treatment with ATO alone only had mild or moderate benefits for the NAFLD rats, while combination of ATO treatment with dietary control did not enhance the effect of dietary control on reducing the levels of serum and hepatic triglyceride and free fatty acid, liver injury, and hepatic steatosis in our experimental system. The present study revealed that dietary control remained a basic therapy for the NAFLD, and to some extent, this was similar to a previous report that antioxidant (alpha-tocopherol plus ascorbic acid) does not increase the efficacy of lifestyle intervention alone in NAFLD model [17]. Apparently, treatment with ATO or antioxidant does not synergize with dietary control in inhibiting HFD-induced liver damage. Our findings were different from that the findings by Martin-Castillo et al, who found that the combination of ATO treatment with a standard diet did reduce the scores of NAFLD activity more [18]. The difference between our and their findings may be due to different models studied.

Previous studies have shown that the transcription factors, SREBP-1c, the PPARα, and the expression of their targeting genes, such as the FAS, ACC, CPT-1, and ACO, are crucial for the development of NAFLD. The SREBP-1c can modulate the expression of a large number of genes involved in the uptake of lipoproteins, the synthesis of cholesterol, TG, and VLDL[19]. The PPARα regulates the expression of the genes involved in mitochondrial and liver fatty acid β-oxidation[20]. Down-regulation and deficient expression of PPARα are associated with the development of NASH, and treatment with agonist for PPARα prevents and inhibits the development of NAFLD[21,22]. Consistently, we found that HFD increased the transcription of SREBP-1c, FAS, and ACC, but decreased the levels of hepatic PPARα mRNA transcripts in the rats while dietary control and treatment with ATO down-regulated the expression of hepatic SREBP-1c and its targeting genes, but up-regulated the transcription of PPARα mRNA in the rats. Our data were consistent with previous findings that treatment with ATO up-regulated the expression of PPARα, liver fatty acid β-oxidation, and reduced the liver TG in rats [23,24]. Therefore, the down-regulated expression of SREBP-1c and its targeting genes that inhibiting lipogenesis, and up-regulated expression of PPARα that promoting lipid metabolism, may contribute to the therapeutic effect of dietary control and ATO treatment on the HFD-induced hyperlipidemia and liver injury in NAFLD rats.

HMG-CoA reductase contains a sterol-sensing domain and is crucial for sterol synthesis, which is negatively regulated by binding to Insig. When plasma sterol levels are low, SREBP is released by the cleavage of a membrane-bound precursor protein and migrates to the nucleus, where it binds to the sterol regulatory element (SRE) and activates the transcription of genes for HMG-CoA reductase and other enzymes involved in the cholesterol synthesis. On the other hand, elevated levels of plasma cholesterol promote the proteolytic cleavage of SREBP from the membrane ceases and protein degradation in the nuclei[25,26]. We found that treatment with ATO at a dose that has been demonstrated to be effective and safe for the control of hyperlipidemia could significantly reduce the hepatic expression of HMG-CoA reductase. More importantly, the relative levels of hepatic HMG-CoA reductase expression in the DCA group were significantly lower than that of the ATO and DA groups of rats. The significantly reduced levels of hepatic HMG-CoA reductase may be associated with the lower levels of serum TC and LDL-C in the DCA group of rats. Our data indicate that combination of ATO treatment with dietary control may be an effective therapeutic strategy for the treatment of HFD-induced hypercholesterolemia and related cardiovascular disease.

## Conclusions

Our data indicated that treatment with ATO had mild or moderate effect on inhibiting the progression of NAFLD and hyerlipidemia in HFD-fed rats. ATO treatment enhanced the effect of dietary control in reducing the levels of serum TC and LDL-C, but not TG, FFA, hepatic lipids and liver steatosis in HFD-fed rats.

## Materials and methods

### Animals and treatment

Fifty-two male Sprague-Dawley (SD) rats at 6 weeks of age and weighing 180-220 g were from the Experimental Animal Center of Guangdong Province (China), and housed in a specific pathogen free facility maintained at a cycle of 12 h light/dark and a constant temperature of 22°C~26°C, and a relative humidity of 65 ± 15%.

The rats were randomized and fed with normal chow diet (n = 14, 10% of calories derived from fat; D12450B) or high fat diet (HFD, n = 38, 60% of calories derived from fat; D12492) for 12 weeks to induce NAFLD with hyperlipidemia, as described previously [11,12]. At the end of the 12-week induction, six rats from normal chow diet group or HFD group were randomly chosen and sacrificed. Their liver histopathology was analyzed for the development of NAFLD. Subsequently, the 32 HFD-fed rats were further randomized into four groups and continually fed with HFD (model group), with HFD and treated P.O with 30 mg/kg of ATO (Sigma-Aldrich, St. Louis, MO) (ATO group), with normal chow diet (dietary control, DC group), or with normal chow diet and treated with the same dose of ATO (combination of dietary control with ATO, DCA group) daily for 8 weeks, respectively. Rats fed with normal chow diet without exposure to HFD were used as the normal controls.

At the end of dietary control and ATO treatment, the rats were fasted for 12 hours and sacrificed. Their blood samples were collected from the abdominal aorta, and their sera were prepared by centrifugation, frozen, and stored at -20°C until analysis. Their livers were frozen in liquid nitrogen and stored at -80°C until gene expression analysis. A portion of the liver from individual rats was fixed overnight in 10% formalin for histological analysis. Two additional samples of liver tissues (150 mg) were stored at -80°C for Westernblot analysis assay and liver lipids measurement. The experimental protocols were approved by the Animal Care and Protection Committee of Sun Yat-Sen University.

### Analyses of serum lipids and transaminases

The concentrations of serum total cholesterol (TC), triglyceride(TG), low density lipoprotein-cholesterol (LDL-C), high density lipoprotein-cholesterol (HDL-C), aspartate aminotransferase (AST), and alanine aminotransferase (ALT) were measured using the corresponding commercial enzyme kits (Biosino, Beijing, China) on an automatic biochemistry analyzer (Olympus AU600,Tokyo, Japan). The levels of serum free fatty acid (FFA) were assayed using a commercial kit, according to the manufacturer's instruction (R&D, Minneapolis, USA).

### Liver triglyceride and cholesterol

Total lipids were extracted from 100 mg of liver tissues, according to the method of Bligh and Dyer [27]. Briefly, total lipids in liver tissues were extracted with chloroform-methanol (2:1). After evaporation overnight, the extracted lipids were re-suspended in 10% triton and isopropanol, and quantified using a commercial enzyme assay kit (Biosino, Beijing, China) on an automatic biochemistry analyzer.

### RNA extraction and quantitative Real-Time Polymerase Chain Reaction (qRT-PCR)

The relative levels of sterol regulatory element binding protein 1c (SREBP-1c), fatty acid synthase (FAS), acetyl-CoA carboxylase (ACC), peroxisome proliferator-activated receptor alpha (PPARα), acyl-CoA oxidase (ACO), carnitine palmitoyltransferase-1 (CPT-1), and mRNA transcripts to control glyceraldehyde-3-phosphate dehydrogenase (GAPDH) were determined by qRT-PCR. Briefly, total RNA was extracted from individual liver samples using Trizol reagent (Invitrogen, USA) and reversely transcribed into cDNA using the super-Transcript kit, according to the manufacturer's protocol (Genecopoeia, USA). Subsequently, the cDNA was used as the template for characterization of the relative levels of mRNA transcripts of individual target genes to control GADPH by qRT-PCR using SYBR Green fluorescence and the specific primers on an iCycler thermocycler (BioRad Hercules, CA). The sequences of the primers were synthesized, according to the previous studies[28,29] and are shown in Table 4. The PCR reactions were performed in duplicate at 95°C for 10 min and subjected to 35 cycles of 95°C for 10 s, 60°C for 20 s, and 72°C for 15 s, followed by extension at 72°C for 10 min. The values of threshold cycle (Ct) were determined by automated threshold analysis using Opticon Monitor 3.1 software. The relative levels of each gene expression were determined by the 2^-ΔΔCt ^method.

**Table 4:** The sequences of primers

mRNA	Forward Primer	Reverse Primer
SREBP-1c	5'-GGA GCCATGGATTGCACATT-3'	5'-AGGAAGGCTTCCAGAGAGGA-3'
FAS	5'-AGGTGCTAGAGGCCCTGCTA-3'	5'-GTGCACAGACACCTTCCC AT-3'
ACC	5'-AGGAAGATGGTGTCCCGCTCTG-3'	5'-GGGGAGATGTGCTGGGTCAT-3'
PPARα	5'-CCC TCTCTCCAGCTTCCAGCCC-3'	5'-CCACAAGCGTCTTCTCAGCCATG-3'
ACO	5'-CTTTCTTGCTTGCCTTCCTTCTCC-3'	5'-GCCGTTTCACCGCCTCGT A-3'
CPT1	5'-GTGCTGGAGGTGGCTTTGGT-3'	5'-TGCTTGACGGATGTGGTTCC-3'
GAPDH	5'- GAACGGGAAGCTCACTGGC -3'	5'- GCATGTCAGATCCACAACGG -3'

### Western blot analysis of HMG-CoA reductase

The expression of hydroxy-3-methylglutaryl coenzyme A (HMG-CoA) reductase

in hepatic tissue was determined by Western blot assay. Briefly, frozen liver samples were homogenized in RIPA lysis buffer containing protease and phosphatase inhibitors. After centrifuged, the protein contents in the lysates were measured using a BCA protein assay kit (Beyotime, China). Individual lysates (30 ug) were separated by 10% SDS-PAGE and electrotransferred to polyvinylidene difluoride (PVDF) membranes. After being blocked with 5% non-fat milk in Tris-buffered saline with 0.1% Tween-20 (TBST, 25 mM Tris, pH 8.0, 137 mM NaCl, 2.7 mM KCl, and 0.1% Tween-20) at room temperature for 60 min, the membranes were incubated with anti- HMG-CoA reductase or anti-GAPDH antibodies (Santa Cruze Biotechnology, Santa Cruze, USA) overnight at 4°C. The bound antibodies were detected with horseradish peroxidase (HRP)-conjugated goat anti-rabbit IgG antibodies (Santa Cruze Biotechnology) and visualized using the enhanced chemiluminescence (ECL, Pierce, Rockford, IL) system and exposed to X-ray film (Kodak, Japan).

### Histological analysis of the liver

After eight weeks of intervention, the rats were sacrificed and their livers were fixed by 10% buffered formalin and embedded in paraffin. The liver sections (5 μm) were stained with hematoxylin and eosin (H&E), and the steatoic degrees of individual liver samples were examined and scored, according to the percentage of hepatocytes containing lipid droplets [30] by a pathologist in a blinded manner.

### Statistical analysis

Values were expressed as mean ± SD. The difference among groups was analyzed by one-way ANOVA and Students t-test. The association among variants was analyzed by the least significant difference (LSD) test using the SPSS 13.0 software. The accepted level of significance was *p *< 0.05.

## Competing interests

The authors declare that they have no competing interests.

## Authors' contributions

ZJ designed the research and revised the manuscript. GJ performed the experiment, assisted in the study design and drafted the manuscript. XZ was responsible for the data collection and interpretation of the results. LL carried out the biochemical analysis and assisted with collecting the data. PL assisted in performing quantitative real-time PCR.

All authors listed have read and approved the final manuscript.
